# Overexpression of the *CaTIP1-1* Pepper Gene in Tobacco Enhances Resistance to Osmotic Stresses

**DOI:** 10.3390/ijms151120101

**Published:** 2014-11-04

**Authors:** Yan-Xu Yin, Shu-Bin Wang, Huai-Juan Xiao, Huai-Xia Zhang, Zhen Zhang, Hua Jing, Ying-Li Zhang, Ru-Gang Chen, Zhen-Hui Gong

**Affiliations:** 1 College of Horticulture, Northwest A&F University, Yangling, Shaanxi 712100, China; E-Mails: yinyanxu2008@nwsuaf.edu.cn (Y.-X.Y.); yuanyi041ban@126.com (H.-J.X.); zhang1142154371@163.com (H.-X.Z.); zhangzhen030@163.com (Z.Z.); jinghua08232014@126.com (H.J.); zyl41982@126.com (Y.-L.Z.); rugangchen@126.com (R.-G.C.); 2 Institute of Vegetable Crops, Jiangsu Academy of Agricultural Sciences, Nanjing, Jiangsu 210014, China; E-Mail: wangsbpep@163.net; 3 College of Food and Biological Engineering, Xuchang University, Xuchang, Henan 461000, China

**Keywords:** osmotic stress, antioxidant enzymes, *Capsicum annuum* L., *CaTIP1-1*, tobacco

## Abstract

Both the gene expression and activity of water channel protein can control transmembrane water movement. We have reported the overexpression of *CaTIP1-1*, which caused a decrease in chilling tolerance in transgenic plants by increasing the size of the stomatal pore. *CaTIP1-1* expression was strongly induced by salt and mannitol stresses in pepper (*Capsicum annuum*). However, its biochemical and physiological functions are still unknown in transgenic tobacco. In this study, transient expression of CaTIP1-1-GFP in tobacco suspension cells revealed that the protein was localized in the tonoplast. *CaTIP1-1* overexpressed in radicle exhibited vigorous growth under high salt and mannitol treatments more than wild-type plants. The overexpression of *CaTIP1-1* pepper gene in tobacco enhanced the antioxidant enzyme activities and increased transcription levels of reactive oxygen species-related gene expression under osmotic stresses. Moreover, the viability of transgenic tobacco cells was higher than the wild-type after exposure to stress. The pepper plants with silenced *CaTIP1-1* in P70 decreased tolerance to salt and osmotic stresses using the detached leaf method. We concluded that the *CaTIP1-1* gene plays an important role in response to osmotic stresses in tobacco.

## 1. Introduction

Water is an essential part of life. Water transport in plants is significantly controlled through integral membrane channel proteins, called aquaporins, which belong to the major intrinsic proteins (MIPs) of a multigene family [1]. Water management is mediated by aquaporins in vascular plants, which is required in different physiological processes [2]. Abiotic stresses, including osmotic stresses (drought and salt stress) and low temperature, can enhance water loss in plants [3,4,5,6,7]. Plants under osmotic stress have lower germination, stunted growth, reduced root function, a slower growth rate and lower yield and, ultimately, die [3,8].

Unlike animals or microbes, plants are known to express a larger number of aquaporins [9,10]. Based on the sequence homology and subcellular localization, the MIP gene family in plants is classified into seven evolutionarily different subfamilies, including the plasma membrane intrinsic proteins (PIPs), the tonoplast intrinsic proteins (TIPs), the nodulin-26-like intrinsic proteins (NIPs), the small basic intrinsic proteins (SIPs), the GlpF-like intrinsic proteins (GIPs), the hybrid intrinsic proteins (HIPs) and the uncategorized X intrinsic proteins (XIPs) [11]. Subcellular localization of TIPs was observed at the tonoplast or plasma membrane [12]. The tonoplast membrane is highly permeable to water. The expression of *GhTIP1-1* is involved in response to low temperatures, which gradually increased in cotyledons under cold stress [13]. The importance of aquaporins in abiotic stresses has been demonstrated through genetic engineering, including reverse genetics and overexpression tools [7,14,15,16,17,18]. The osmotic water permeability coefficient was increased in the *Arabidopsis* leaf mesophyll (transient gene expression). Overexpressing the *SlTIP2-2* gene also enhanced the relative transpiration compared to the control under water stress [14]. Conversely, PIP1b overexpression tobacco plants required more water consumption to maintain a normal phenotype [19]. Two alternative mechanisms have been developed to explain the performance of aquaporins in transgenic plants under water stress [20]. The overexpression of the plasma membrane intrinsic protein (PIP) subfamily in tobacco increased drought or salt tolerance by enhancing the antioxidant system [21,22,23]. It is necessary to address the function of aquaporins involved in the regulation of osmotic stress [24].

There are certain limitations in the genetic transformation of *Capsicum* biotechnology due to the low efficiency of pepper’s regeneration ability [25]. The virus-induced gene-silencing (VIGS) method is an effective tool to study gene functions in different tissues [26,27]. In our previous study, we found that overexpression of *CaTIP1-1* (*CaAQP*, Accession No. GU116569) decreased chilling tolerance in transgenic tobacco plants by increasing the stomatal aperture. The expression patterns of *CaTIP1-1* were different between low temperature and osmotic stress in pepper seedlings [7]. However, the role of *CaTIP1-1* in pepper plants under low temperature and osmotic stress is unclear. In this study, we reported the subcellular localization of *CaTIP1-1* and determined antioxidant enzyme activities related to gene expression during abiotic stresses. The present study revealed that overexpression of the *CaTIP1-1* pepper gene conferred tolerance to osmotic stresses in plants, which is correlated to increasing antioxidant enzyme activities and cell viability under stresses. Finally, we have used *CaTIP1-1* gene silencing as a VIGS tool to analyze the function in pepper under osmotic stresses.

## 2. Results and Discussion

### 2.1. Subcellular Localization of CaTIP1-1

**Figure 1:**
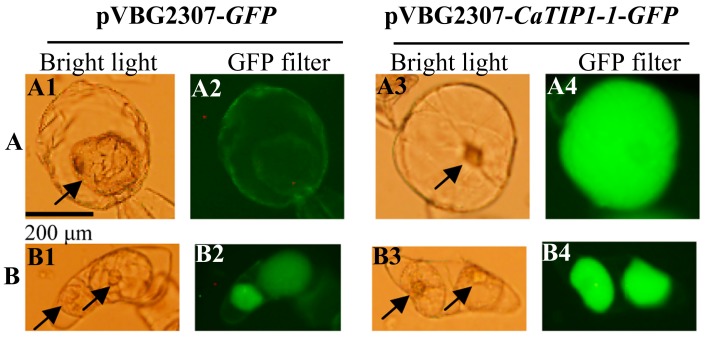
Subcellular localization of CaTIP1-1. A fluorescent image was taken with a fluorescent microscope (BX51; Olympus) and a 50-W mercury lamp. (**A**) Control (untreated) suspension-cultured cells of tobacco and (**B**) suspension-cultured cells of tobacco were plasmolysed in 0.8 M mannitol for 15 min. The arrow indicates the nucleus; (**A1**,**A3**,**B1**,**B3**) image taken by a fluorescent microscope (bright light); (**A2**,**A4**,**B2**,**B4**) image taken by a fluorescent microscope (GFP filter).

Overexpressed GFP and GFP-fused CaTIP1-1 (CaTIP1-1-GFP) were reinserted into the vector pVBG2307. Suspension-cultured cells of tobacco were plasmolysed in 0.8 M mannitol for 15 min and compared with the control. In order to investigate the subcellular localization of CaTIP1-1 in treated and control cells, a fluorescent image was taken with a fluorescent microscope (BX51; Olympus, Tokyo, Japan) and a 50-W mercury lamp. The results in Figure 1A2,B2, showed that pVBG2307-*GFP* protein was expressed in the cell membrane and nucleus in the case of the control treatment, while the protein of treated cells was expressed in cell nucleus, cytoplasm and plasma membrane. It is clear from Figure 1A4,B4 that the subcellular localization of pVBG2307-*CaTIP1-1*-*GFP* was expressed inside the cell in the case of treated cells, and there was no expression in the cell wall; while, in the control treatment, the expression was inside the cell. These results suggest that CaTIP1-1 was localized in the tonoplasts.

### 2.2. Radicle Growth of CaTIP1-1-Overexpressing Tobacco Plants

**Figure 2:**
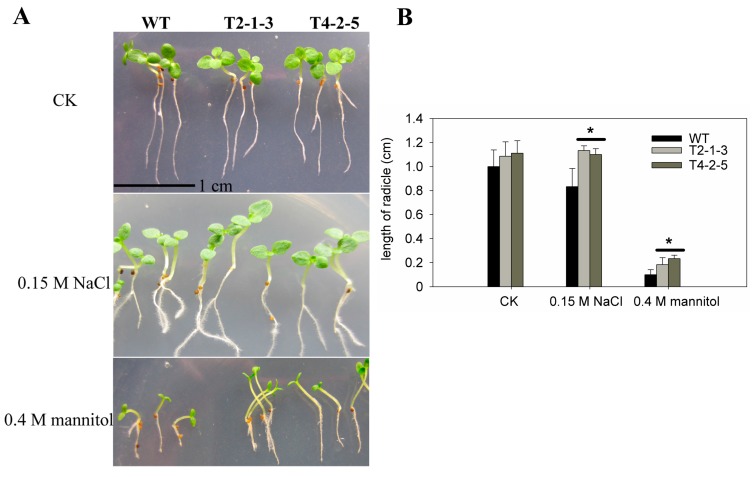
Seedling growth of WT and transgenic lines of T3 tobacco plants subjected to osmotic stress. (**A**) Germinated seedlings with radicles of similar length were cultured in half-strength MS medium supplemented with 0.15 M NaCl or 0.4 M mannitol or control, and photographs were taken after 7 days of incubation; the scale bar represents 1.0 cm; (**B**) Results are the mean ± standard error (SE), replicated thrice. * indicates significant differences when compared with the control at a *p* value <0.05.

Tobacco plants were transformed with a full-length sequence of the *CaTIP1-1* pepper gene driven by the constitutive CaMV 35S promoter. Two lines (T2-1-3 and T4-2-5) were selected for further analysis. Both the transgenic and WT plants have similar radicle growth under normal growth conditions (Figure 2A,B). All transgenic seedlings treated with 0.15 M NaCl showed normal root and cotyledon growth, which was the same as the salt-free medium. While the radicle length of the WT seedlings decreased by 26.5% when compared to the transgenic plants (Figure 2A,B). After 7 days when exposed to the higher mannitol concentration, the transgenic plant seedlings’ growth quantity was two-fold more than that of the WT seedlings (Figure 2A,B).

### 2.3. Analysis of Relative Water Content (RWC), Relative Electrolyte Leakage (REL), Malondialdehyde (MDA) and Antioxidant Enzyme Activities

**Figure 3:**
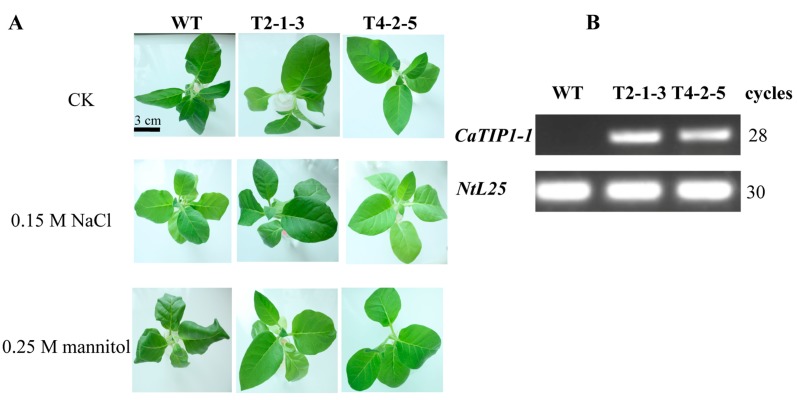
Phenotype analysis and expressions of *CaTIP1-1* in WT and transgenic plants. (**A**) Phenotype analysis of WT and T3 transgenic plants (T2-1-3 and T4-2-5 are independent transgenic lines) that were subjected to salt (0.15 M NaCl) or mannitol (0.25 M) stress for 12 h. The scale bar represents 3.0 cm; (**B**) Expressions of *CaTIP1-1* in WT and transgenic plants (T2-1-3 and T4-2-5 are independent transgenic lines) under normal growth conditions by semi-quantitative PCR. PCR was performed for 28 or 30 cycles for different genes.

**Figure 4:**
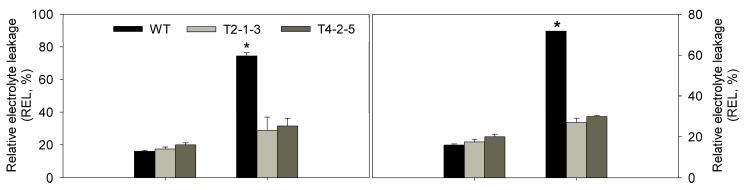

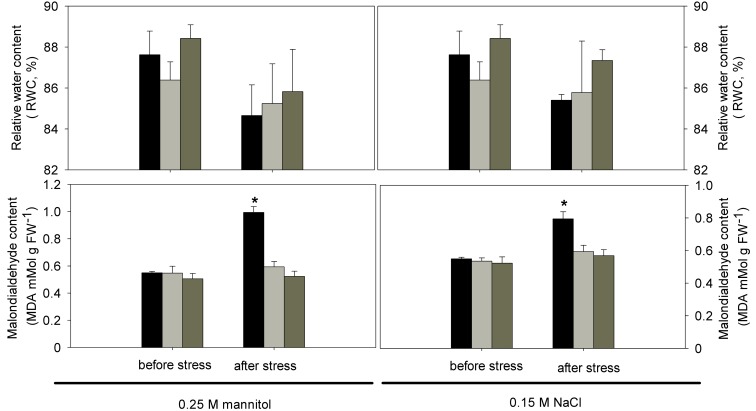
Physiological analysis of WT and *CaTIP1-1-*overexpressing transgenic lines under normal conditions and osmotic stress. Changes in relative water content (RWC), relative electrolyte leakage (REL) and malondialdehyde (MDA) were measured in the leaves of WT and *CaTIP1-1-*overexpressing transgenic lines (T2-1-3 and T4-2-5, which are independent transgenic lines) under normal conditions or osmotic stresses (0.15 M NaCl or 0.25 M mannitol for 12 h). The results are the mean ± standard error (SE), replicated thrice. * indicates significant difference when compared with the control at a *p* value <0.05.

The T3 tobacco seedlings were subjected to osmotic stresses. Under these conditions, in the WT tobacco leaves, wilting became evident after 12 h, which was earlier than that of the transgenic plants (Figure 3A). To investigate the transcription levels of *CaTIP1-1*, transgenic lines were obtained and confirmed by PCR analysis using genomic DNA (data not shown); the expression was firstly detected in the WT and transgenic plants (Figure 3B) and then evaluated by qRT-PCR under the osmotic stresses. The differences between the WT and transgenic plants related to the relative water content (RWC), relative electrolyte leakage (REL), malondialdehyde (MDA) and antioxidant enzyme activities were measured at various times (data for after 12 h only included). The MDA content was higher in the WT plants than in the transgenic lines after exposure to the osmotic stresses (Figure 4). After 12 h of osmotic stress, the WT displayed a greater increase in REL than transgenic plants. Both lines of transgenic plants gave similar REL values, which were close to that in WT before the stress treatments (Figure 4). The RWC displayed non-significant differences in the treatments (Figure 4).

**Figure 5:**
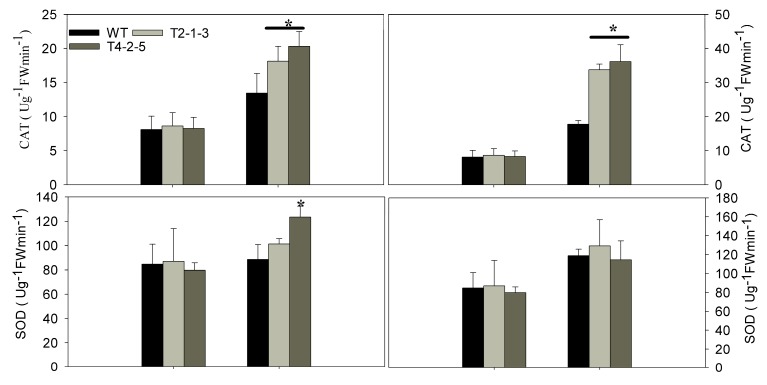

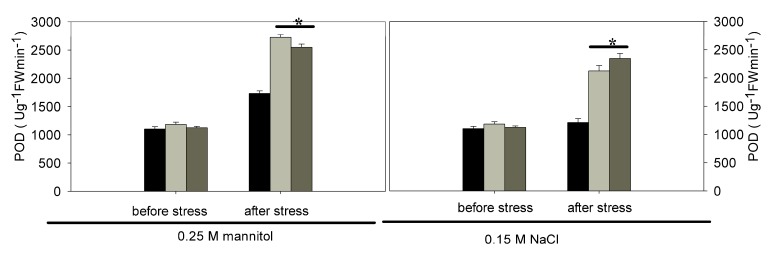
SOD, catalase (CAT) and peroxiredoxin (POD) were detected in the leaves of the transgenic lines (T2-1-3 and T4-2-5) and WT at 12 h after osmotic stress. Results are the mean ± standard error (SE), replicated thrice. * indicates significant differences when compared with the control at a *p* value <0.05.

The activities of three antioxidant enzymes (CAT, SOD and POD) were assessed in leaves during the different stages of osmotic stress. Under normal growth conditions, the stresses had minimal effects on the SOD activities from the transgenic plants, except for the line of T4-2-5 that increased 39.23% from 88.64 to 123.42 U/gFW/min (0.25 M mannitol stress). In addition, the transgenic lines had greatly improved CAT and POD activities under the osmotic stresses (Figure 5).

### 2.4. Cell Viability under Osmotic Stresses

**Figure 6:**
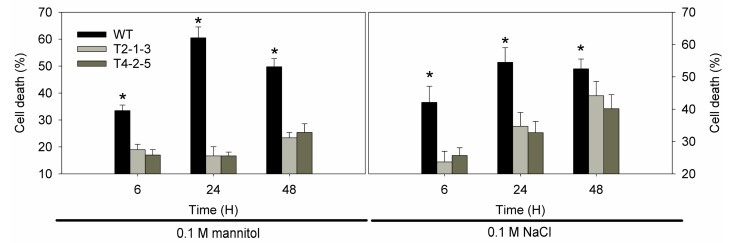
Cell death was determined by fluorescein diacetate (FDA) staining in the tobacco cell suspension induced by 0.1 M mannitol or NaCl for 6, 24 and 48 h. Results are the mean ± standard error (SE), replicated thrice. * indicates significant differences compared with the control at a *p* value <0.05.

The treatments with 0.1 M NaCl and 0.1 M mannitol significantly decreased the viability of the tobacco suspension cells (76.51% and 55.9%, respectively) for 6 h (Figure 6). However, the same concentration of mannitol caused a slight increase in the death rate (T) at 48 h compared to the control at 6 h. The cell death rate was higher in salt stress than mannitol stress. Overexpression of *CaTIP1-1* led to a marked decrease in the cell death rate under osmotic stress. It was also clear that the cell viability percent in transgenic plants was higher than wild-type plants.

### 2.5. ROS-Related Genes Expression under Osmotic Stresses in Transgenic Tobacco

**Figure 7:**
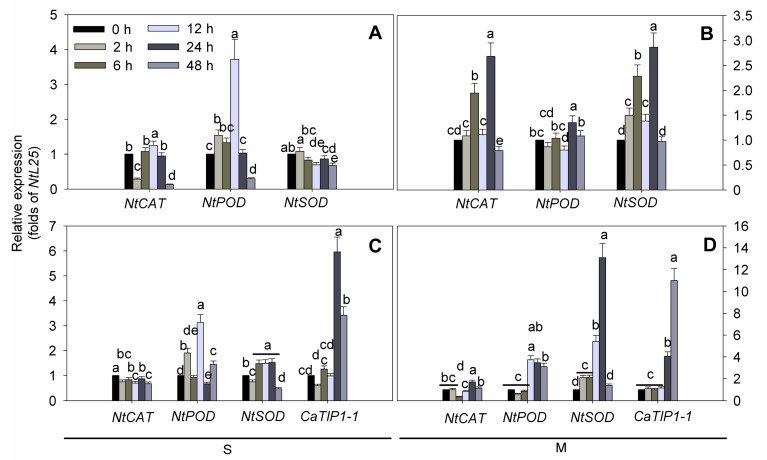
Quantitative RT-PCR was used to assess the transcript levels of *CaTIP1-1*, *NtCAT*, *NtSOD* and *NtPOD* in the leaves of the transgenic lines and the wild-type. The relative fold differences in the mRNA levels were calculated using the 2^−∆∆*C*t^ formula with *NtL25* as the internal control. Bars with different lower case letters in each group were significantly different, as determined using Duncan’s multiple range test (*p* < 0.05). S indicates 0.15 M NaCl stress ((**A**) wild-type and (**C**) transgenic plants); M means 0.25 M mannitol stress ((**B**) wild-type and (**D**) transgenic plants).

Under osmotic stress conditions, the transcription level of *CaTIP1-1* remained higher in the transgenic (T) plants than in WT (Figure 7). Furthermore, under osmotic stress, the transcription levels of *CaTIP1-1*, *NtCAT*, *NtSOD* and *NtPOD* were assessed in the two transgenic lines. The two lines showed similar results; therefore, only one line is presented (Figure 7). The transcription levels of *NtCAT*, *NtSOD* and *NtPOD* were downregulated when the WT plants were subjected to osmotic stress for 48 h (Figure 7A,C). A rapid accumulation of the *NtSOD* transcript in transgenic plants was observed within 2 h (2.1-fold); it peaked at 24 h (13.1-fold) and reached a normal level (1.4-fold) 48 h after mannitol treatment. The transcription levels of *NtPOD* were higher in the transgenic plants than the WT plants.

### 2.6. Leaf Discs Phenotype of Gene-Silenced Pepper in Response to Osmotic Stresses

**Figure 8:**
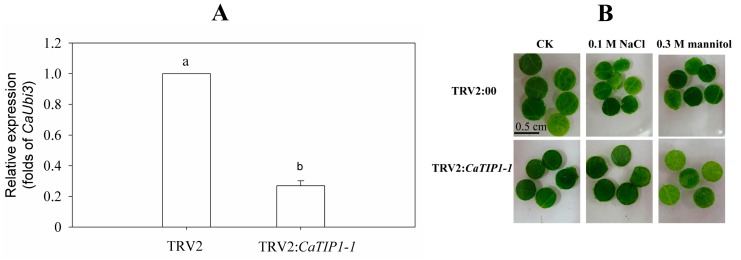
*CaTIP1-1* expression level of gene-silenced pepper plants. (**A**) The expression of *CaTIP1-1* in gene-silenced pepper (TRV2:*CaTIP1-1*) cv P70 and control plants (TRV2:00) were tested at 45 days after inoculation; (**B**) leaf discs phenotypes (0.5 cm in diameter) of the gene-silenced plants in response to 0.1 M salt stress and 0.3 M mannitol stress after 48 h.

When the positive control (inoculated with TRV2:*CaPDS*) showed a large bleaching symptom, young leaves of *CaTIP1-1*-silenced plants and the negative control (inoculated with TRV2) were collected to detect the silencing effect. Compared to the negative control, the *CaTIP1-1* silencing rate reached nearly 75% (Figure 8A). Leaf discs (0.5 cm in diameter) were obtained from the young leaves with a rate of more than 50% gene silencing to perform osmosis stress. The *CaTIP1-1*-silenced materials led to quick chlorophyll degradation after 48 h (Figure 8B).

### 2.7. Discussion

TIPs’ subcellular localization has been performed by immunofluorescence and the transient or stable expression of fluorescent protein fusions [28]. The tobacco suspension cell technique is suitable to observe the localization of modified GFPs with various signals in different compartments in a vacuolar-sorting system [29,30]. Based on its amino acid sequence, CaTIP1-1 is predicted to be a TIP and to be aligned together with the 35 AQPs of *Arabidopsis* [31]. In the current results, the CaTIP1-1-GFP protein was investigated in the central vacuole of the suspension cells using the transient expression system [32]. The massive amount of TIPs can facilitate water movement across the cells, which might favor the growth of transgenic plants under normal conditions. However, *CaTIP1-1* overexpression showed no significant effect on the transgenic plants without stress. These results suggest that environmental stimuli regulated the expression of *CaTIP1-1* and *PgTIP* at different levels [16]. *Arabidopsis* mutant lines *attip1-1* and *attip1-2* are alive and showed no lethal phenotype [33]. The pepper plants with silenced *CaTIP1-1* in P70 decreased in their tolerance to salt and mannitol stresses.

It has been shown previously that *CaTIP1-1* has a negative role in chilling stress. This also indicated that the mechanisms of overexpression of *CaTIP1-1* decreased the water use efficiency due to stomatal aperture changes when exposed to low temperatures [7]. The expression analysis of the *CaTIP1-1* gene showed upregulation at a low salt and mannitol concentration in pepper seedlings [7]. Osmotic stress significantly increased the *CaTIP1-1* gene expression in transgenic plants, which showed higher RWC. It is unclear whether the beneficial effects are related to water transport or have a well-established role in the membranes that have been damaged by oxidative stress, which in this role caused less electrolyte leakage.

Plants commonly accumulate reactive oxygen species (ROS) in response to environmental stresses [34], and ROS accumulation during stress greatly depends on the balance between ROS production and ROS scavenging [35]. The present study considered the possible regulation role of ROS-scavenging enzymes, including superoxide dismutase (SOD), catalase (CAT) and peroxiredoxin (POD). The current results indicated that the ROS-scavenging enzymes’ activities were increased in both WT and transgenic plants when exposed to osmotic stress. Controlling the ROS balance was shown to be important for abiotic and biotic stresses tolerance, such as drought, salinity stress and *Phytophthora capsici* infection, in transgenic plants or tolerant cultivars [36]. In addition, the higher activities of CAT and POD during stress may be responsible for reducing the damage of transgenic plants. At the same time, our results indicated that the transcription of *CaTIP1-1* increased after 12 h. Macroarray experiments revealed PIP2 and TIP1 homology that showed 20% to 40% decreases in abundance 6 h after treatment [3]. These mechanisms may act in concert with the overexpression of *CaTIP1-1* in tobacco seedlings. 

Generally, plant growth and root development stages are sensitive to osmotic stress. The radicles of transgenic plants were more tolerant to osmotic stresses than those of WT plants. The present data also showed that the transgenic tobacco seedlings exhibited less damage, which means that the overexpression of *CaTIP1-1* in plants makes them more tolerant to osmotic stresses. The WT plants wilted within 12 h of treatment with 0.15 M NaCl or 0.25 M mannitol. The transcription levels of *CaTIP1-1* showed high levels in the transgenic plants after 6 h. The expression levels of *CaTIP1-1* may be related to the tolerance to osmotic stresses. The complex expression patterns of aquaporins in plants have been determined by different groups under various environmental conditions [9,14,37]. In the current study, the transgenic plants preserved the RWC at a constant level. It is possible that *CaTIP1-1* overexpression influences the activities of other endogenous aquaporins, which positively regulates the response to the stress [37]. Further research is needed to completely understand the expression of other endogenous aquaporins under osmotic stress. MDA and REL are important indicators of cell membrane injury. Osmotic stress significantly increased the MDA and REL in the WT, which suggested that the WT plants suffered more damage than the transgenic plants. The cell death rate also displayed similar results.

## 3. Experimental Section

### 3.1. Plant Materials, Cell Culture and Seedling Treatment

Tobacco (*Nicotiana tabacum*) cv Bairihong was used for gene transformation according to the leaf disc method; T3 transgenic tobacco plants were used for all experiments, and the seedlings were selected and incubated as previously described [7]. Callus cultures and subcultures were maintained on the same medium [38]. The cell suspension cultures of tobacco were cultured in liquid MS medium supplemented with 0.2 mg/L 2,4-dichlrophenoxyacetic acid and 3% sucrose on a rotary shaker. Cells were transferred to fresh medium every week. For the suspension of cultured cells during osmotic stress treatments, fresh cells were transferred to medium containing a high concentration of salt (0.1 M NaCl) or mannitol (0.1 M mannitol). Both wild-type (WT) and transgenic tobacco germinated seeds were exposed to osmotic stresses (0.15 M NaCl or 0.4 M mannitol), and their growth rate was measured. For osmotic and salt stress treatments, ten-week old tobacco seedlings were cultured with 1/2-strength Hoagland’s nutrient solution [39] and then treated with 0.15 M NaCl and 0.25 M mannitol, respectively. Control seedlings were grown in a growth chamber at 25 °C under fluorescent lighting (14 h light/10 h dark cycle, 200 µmol/m^2^/s, 70% relative humidity). Leaves from stress-treated plants were collected at 0, 2, 6, 12, 24 and 48-h intervals after treatment and immediately frozen in liquid nitrogen, then kept at −80 °C for RNA isolation.

### 3.2. Subcellular Localization of CaTIP1-1 Protein

The full length of the *CaTIP1-1* pepper gene coding region was cloned into a pVBG2307 vector [40]. Under the control of a CaMV 35S promoter and fused in the 3' region with the green fluorescence protein (*GFP*) gene, pVBG2307-*CaTIP1-1-GFP* was produced. The specific gene primers are presented in Table 1. The plasmids of pVBG2307-*GFP* (used as the control) and pVBG2307-*CaTIP1-1-GFP* were transformed into tobacco cell suspension culture using *Agrobacterium tumefaciens* by the co-cultivation method [32]. The CaTIP1-1 protein was localized subcellularly using a fluorescent microscope. Plasmolysed cells were examined with a fluorescent microscope (BX51; Olympus) using a 50-W mercury lamp. Suspension-cultured cells of tobacco were plasmolysed in 0.8 M mannitol for 15 min [27].

**Table 1:** Primers used in this investigation.

Primers	Primer Sequence (5'→3')	Explanation
*CaTIP1-1*	F1: CGATGGCGTCACTACTCCTC R1: TGATGTACAGAAGTCCCCTG	RT-PCR
	^a^ F: GC 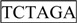 CTCTTCAGTTTGGTTGTAGGC ^b^ R: CGG 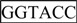 GCACCGAAAGTAACAGCAG	VIGS Vector construct
	^a^ F2: GC 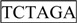 ATGCCGATCCGCCAAATTG ^b^ R2: CGG 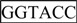 AAAATCTCCTCCACTTGGGATTTGC	Subcellular localization
*GFP*	F: GTAAGGGAGAAGAACTTTTCACTG R: TGTGGTCTCTCTTTTCGTTGG	
*NtCAT*	F: GTATTGCTTGAGGATTACCATTT R: CTTGACAGCAAACCCACG	RT-PCR
*NtSOD*	F: CTCCTACCGTCGCCAAAT R: GCCCAACCAAGAGAACCC	
*NtPOD*	F: GCTGTTCGACGAGTTGTTAACAG R: CTCTGGCTGAGTTGTTGTTGG	
*NtL25*	F: CCTAAAGTATCCCCTCACCACAG R: CTTTCTTCGTCCCATCAGGC	
*CaUbi3*	F: TGTCCATCTGCTCTCTGTTG R: CACCCCAAGCACAATAAGAC	

^a^ Black frames indicate that the primers of *CaTIP1-1*F and *CaTIP1-1*F2 carried the *XbaI* enzyme site; ^b^ black frames indicate that the primers of *CaTIP1-1*R and *CaTIP1-1*R2 carried the *KpnI* enzyme site.

### 3.3. RWC, REL, MDA and Antioxidant Enzyme Assays in the Transgenic Tobacco Plants

Leaves from stress-treated plants were collected 12 h after the stress treatment, and the relative water content (RWC) and relative electrolyte leakage (REL) were measured according to the method of Yin *et al.* [7]. The level of lipid peroxidation was measured in terms of malondialdehyde (MDA) and antioxidative enzymes, followed the procedure of Guo *et al.* [41].

### 3.4. RNA Isolation and Quantitative Real-Time PCR Analysis

Reverse transcription was performed using the Primescript™ first strand cDNA Synthesis Kit (TaKaRa, Dalian, China). Quantitative real-time PCR (qRT-PCR) was performed according to the method of Guo *et al.* [41], and semi-quantitative PCR was carried out as described by Yin *et al.* [7]. The L25 ribosomal protein (*NtL25*, Accession No. L18908) was used as a tobacco reference gene [42]. The ubiquitin-conjugating protein (*CaUbi3*, Accession No. AY486137) was used as a pepper reference gene [43]. The specific gene primers used, including *NtCAT* (Accession No. HF564632), *NtSOD* (Accession No. AB093097) and *NtPOD* (Accession No. AB178953), are presented in Table 1. The relative fold difference in mRNA levels was calculated using the 2^−∆∆*C*t^ formula with *NtL25* as the internal control [22].

### 3.5. Determination of Viability of Tobacco Suspension Cells under Osmotic Stresses

Suspension cells of T3 transgenic tobacco (*Nicotiana tabacum*) cv Bairihong and WT were placed in the culture media amended with 0.1 M NaCl or 0.1 M mannitol to induce salt or osmotic stresses. Control cells were cultured on the same media without adding NaCl or mannitol. Cells were stained with fluorescein diacetate (FDA) (AAT Bioquest, Sunnyvale, CA, USA) to estimate cell viability after stress exposure [44]. Dimethyl sulfoxide (Sigma Aldrich, Saint Louis, MO, USA) was selected as the permeabilizing agent for FDA staining. FDA-stained cells were examined with a fluorescent microscope (BX51; Olympus) using a 50-W mercury lamp. For each sample, at least 200 cells were counted in each treatment.

### 3.6. VIGS Assay of CaTIP1-1 in Pepper Plants

The TRV-based VIGS system was used for gene silencing, as described previously [26]. To generate the *CaTIP1-1*/TRV2 construct, a 325-bp fragment of the *CaTIP1-1* gene was PCR amplified from pepper. The resulting product was cloned into the TRV2 vector using the double digested method with enzymes of *XbaI* and *KpnI*. *Agrobacterium tumefaciens* strain GV3101 harboring pTRV1 was respectively mixed with pTRV2 (as the negative control), TRV2-*CaPDS* (as the positive control) or TRV2-*CaTIP1-1* at a 1:1 ratio. The mixtures were inoculated into pepper cv P70 at the fully-expanded cotyledons stage. After injection, all of the seedlings were placed at 18 °C and 60% relative humidity for 2 days and then moved to a growth chamber according to the protocol [26]. The gene-silenced leaves discs were used for 0.1 M salt or 0.3 M mannitol stress.

### 3.7. Statistical Analysis

Statistical analysis was performed using the Statistical Analysis System software (SAS 8.2, North Carolina State University, Cary, NC, USA), and the means were compared using Duncan’s multiple range test, taking *p* < 0.05 as a significant difference. Values were expressed as the mean ± standard error (SE). All experiments were performed and analyzed separately with three biological replicates.

## 4. Conclusions

In conclusion, the overexpression of *CaTIP1-1* pepper gene increased the tolerance in tobacco to osmotic stresses during the seedling stage. The *CaTIP1-1*-transgenic plants showed higher cell viability and antioxidant enzyme activities under osmotic stress compared to the wild-type cells. After exposure to osmotic stresses, in the transgenic plants, there was a rapid accumulation of antioxidant enzyme-related gene transcripts. *CaTIP1-1*-silenced pepper led to quick chlorophyll degradation. We conclude that the overexpression of *CaTIP1-1* pepper gene in tobacco increased osmotic stress tolerance by contributing to the ROS balance.

## References

[1] Maurel C., Verdoucq L., Luu D.T., Santoni V. (2008). Plant aquaporins: Membrane channels with multiple integrated functions. Annu. Rev. Plant Biol..

[2] Chaumont F., Tyerman S.D. (2014). Aquaporins: Highly regulated channels controlling plant water relations. Plant Physiol..

[3] Boursiac Y., Chen S., Luu D.T., Sorieul M., van den Dries N., Maurel C. (2005). Early effects of salinity on water transport in *Arabidopsis* roots. Molecular and cellular features of aquaporin expression. Plant Physiol..

[4] Li G.W., Peng Y.H., Yu X., Zhang M.H., Cai W.M., Sun W.N., Su W.A. (2008). Transport functions and expression analysis of vacuolar membrane aquaporins in response to various stresses in rice. J. Plant Physiol..

[5] Wang X., Li Y., Ji W., Bai X., Cai H., Zhu D., Sun X.L., Chen L.J., Zhu Y.M. (2011). A novel *Glycine soja* tonoplast intrinsic protein gene responds to abiotic stress and depresses salt and dehydration tolerance in transgenic *Arabidopsis thaliana*. J. Plant Physiol..

[6] Pou A., Medrano H., Flexas J., Tyerman S.D. (2013). A putative role for TIP and PIP aquaporins in dynamics of leaf hydraulic and stomatal conductances in grapevine under water stress and re-watering. Plant Cell Environ..

[7] Yin Y.X., Guo W.L., Zhang Y.L., Ji J.J., Xiao H.J., Yan F., Zhao Y.Y., Zhu W.C., Chen R.G., Chai W.G. (2014). Cloning and characterisation of a pepper aquaporin, *CaAQP*, which reduces chilling stress in transgenic tobacco plants. Plant Cell Tissue Organ Cult..

[8] Munns R. (2002). Comparative physiology of salt and water stress. Plant Cell Environ..

[9] Venkatesh J., Yu J.W., Park S.W. (2013). Genome-wide analysis and expression profiling of the *Solanum tuberosum* aquaporins. Plant Physiol. Biochem..

[10] Reuscher S., Akiyama M., Mori C., Aoki K., Shibata D., Shiratake K. (2013). Genome-wide identification and expression analysis of aquaporins in tomato. PLoS One.

[11] Danielson J.A.H., Johanson U. (2008). Unexpected complexity of the aquaporin gene family in the *moss Physcomitrella patens*. BMC Plant Biol..

[12] Gattolin S., Sorieul M., Frigerio L. (2011). Mapping of tonoplast intrinsic proteins in maturing and germinating *Arabidopsis* seeds reveals dual localization of embryonic TIPs to the tonoplast and plasma membrane. Mol. Plant.

[13] Li D.D., Tai F.J., Zhang Z.T., Li Y., Zheng Y., Wu Y.F., Li X.B. (2009). A cotton gene encodes a tonoplast aquaporin that is involved in cell tolerance to cold stress. Gene.

[14] Sade N., Vinocur B.J., Diber A., Shatil A., Ronen G., Nissan H., Wallach R., Karchi H., Moshelion M. (2009). Improving plant stress tolerance and yield production: Is the tonoplast aquaporin *SlTIP2;2* a key to isohydric to anisohydric conversion?. New Phytol..

[15] Liu C.W., Fukumoto T., Matsumoto T., Gena P., Frascaria D., Kaneko T., Katsuhara M., Zhong S.H., Sun X.L., Zhu Y.M. (2013). Aquaporin *OsPIP1;1* promotes rice salt resistance and seed germination. Plant Physiol. Biochem..

[16] Peng Y.H., Lin W.L., Cai W.M., Arora R. (2007). Overexpression of a *Panax ginseng* tonoplast aquaporin alters salt tolerance, drought tolerance and cold acclimation ability in transgenic *Arabidopsis* plants. Planta.

[17] Leitao L., Prista C., Moura T.F., Loureiro-Dias M.C., Soveral G. (2012). Grapevine aquaporins: Gating of a tonoplast intrinsic protein (TIP2;1) by cytosolic pH. PLoS One.

[18] Beebo A., Thomas D., Der C., Sanchez L., Leborgne-Castel N., Marty F., Schoefs B., Bouhidel K. (2009). Life with and without *AtTIP1;1*, an *Arabidopsis* aquaporin preferentially localized in the apposing tonoplasts of adjacent vacuoles. Plant Mol. Biol..

[19] Aharon R., Shahak Y., Wininger S., Bendov R., Kapulnik Y., Galili G. (2003). Overexpression of a plasma membrane aquaporin in transgenic tobacco improves plant vigor under favorable growth conditions but not under drought or salt stress. Plant Cell.

[20] Hussain S.S., Iqbal M.T., Arif M.A., Amjad M. (2011). Beyond osmolytes and transcription factors: Drought tolerance in plants via protective proteins and aquaporins. Biol. Plant..

[21] Hu W., Yuan Q.Q., Wang Y., Cai R., Deng X.M., Wang J., Zhou S.Y., Chen M.J., Chen L.H., Huang C. (2012). Overexpression of a wheat aquaporin gene, *TaAQP8*, enhances salt stress tolerance in transgenic tobacco. Plant Cell Physiol..

[22] Zhou S.Y., Hu W., Deng X.M., Ma Z.B., Chen L.H., Huang C., Wang C., Wang J., He Y.Z., Yang G.X. (2012). Overexpression of the wheat aquaporin gene, *TaAQP7*, enhances drought tolerance in transgenic tobacco. PLoS One.

[23] Zhang Y.X., Wang Z., Chai T.Y., Wen Z.S., Zhang H.M. (2008). Indian mustard aquaporin improves drought and heavy-metal resistance in tobacco. Mol. Biotechnol..

[24] Ruiz-Lozano J.M., Porcel R., Azcon C., Aroca R. (2012). Regulation by arbuscular mycorrhizae of the integrated physiological response to salinity in plants: New challenges in physiological and molecular studies. J. Exp. Bot..

[25] Kothari S.L., Joshi A., Kachhwaha S., Ochoa-Alejo N. (2010). Chilli peppers-A review on tissue culture and transgenesis. Biotechnol. Adv..

[26] Wang J.E., Li D.W., Gong Z.H., Zhang Y.L. (2013). Optimization of virus-induced gene silencing in pepper (*Capsicum annuum* L.). Genet. Mol. Res..

[27] Xiao H.J., Yin Y.X., Chai W.G., Gong Z.H. (2014). Silencing of the *CaCP* gene delays salt- and osmotic-induced leaf senescence in *Capsicum annuum* L.. Int. J. Mol. Sci..

[28] Gattolin S., Sorieul M., Frigerio L. (2010). Tonoplast intrinsic proteins and vacuolar identity. Biochem. Soc. Trans..

[29] Mitsuhashi N., Shimada T., Mano S., Nishimura M., Hara-Nishimura I. (2000). Characterization of organelles in the vacuolar-sorting pathway by visualization with GFP in tobacco BY-2 cells. Plant Cell Physiol..

[30] Lee O.R., Cho H.T. (2012). Cytoplasm localization of aminopeptidase M1 and its functional activity in root hair cells and BY-2 cells. Mol. Biol. Rep..

[31] Johanson U., Karlsson M., Johansson I., Gustavsson S., Sjovall S., Fraysse L., Weig A.R., Kjellbom P. (2001). The complete set of genes encoding major intrinsic proteins in *arabidopsis* provides a framework for a new nomenclature for major intrinsic proteins in plants. Plant Physiol..

[32] An G. (1985). High efficiency transformation of cultured tobacco cells. Plant Physiol..

[33] Schussler M.D., Alexandersson E., Bienert G.P., Kichey T., Laursen K.H., Johanson U., Kjellbom P., Schjoerring J.K., Jahn T.P. (2008). The effects of the loss of TIP1;1 and TIP1;2 aquaporins in *Arabidopsis thaliana*. Plant J..

[34] Ben Rejeb K., Abdelly C., Savoure A. (2014). How reactive oxygen species and proline face stress together. Plant Physiol. Biochem..

[35] Mittler R., Vanderauwera S., Gollery M., van Breusegem F. (2004). Reactive oxygen gene network of plants. Trends Plant Sci..

[36] Wang J.E., Liu K.K., Li D.W., Zhang Y.L., Zhao Q., He Y.M., Gong Z.H. (2013). A novel peroxidase *CanPOD* gene of pepper is involved in defense responses to *phytophtora capsici* infection as well as abiotic stress tolerance. Int. J. Mol. Sci..

[37] Jang J.Y., Kim D.G., Kim Y.O., Kim J.S., Kang H. (2004). An expression analysis of a gene family encoding plasma membrane aquaporins in response to abiotic stresses in *Arabidopsis thaliana*. Plant Mol. Biol..

[38] Mimura T., Kura-Hotta M., Tsujimura T., Ohnishi M., Miura M., Okazaki Y., Mimura M., Maeshima M., Washitani-Nemoto S. (2003). Rapid increase of vacuolar volume in response to salt stress. Planta.

[39] Del Amor F.M., Rubio J.S. (2009). Effects of antitranspirant spray and potassium: calcium: magnesium ratio on photosynthesis, nutrient and water uptake, growth, and yield of sweet pepper. J. Plant Nutr..

[40] Ahmed S.S., Gong Z.H., Ji J.J., Yin Y.X., Xiao H.J., Khan M.A., Rehman A., Ahmad I. (2012). Construction of the intermediate vector pVBG2307 by incorporating vital elements of expression vectors pBI121 and pBI221. Genet. Mol. Res..

[41] Guo W.L., Chen R.G., Gong Z.H., Yin Y.X., Ahmed S.S., He Y.M. (2012). Exogenous abscisic acid increases antioxidant enzymes and related gene expression in pepper (*Capsicum annuum*) leaves subjected to chilling stress. Genet. Mol. Res..

[42] Schmidt G.W., Delaney S.K. (2010). Stable internal reference genes for normalization of real-time RT-PCR in tobacco (*Nicotiana tabacum*) during development and abiotic stress. Mol. Genet. Genomics.

[43] Wan H.J., Yuan W., Ruan M.Y., Ye Q.J., Wang R.Q., Li Z.M., Zhou G.Z., Yao Z.P., Zhao J., Liu S.J. (2011). Identification of reference genes for reverse transcription quantitative real-time PCR normalization in pepper (*Capsicum annuum* L.). Biochem. Biophys. Res. Commun..

[44] Sun J.A., Li L.S., Liu M.Q., Wang M.J., Ding M.Q., Deng S.R., Lu C.F., Zhou X.Y., Shen X., Zheng X.J. (2010). Hydrogen peroxide and nitric oxide mediate K^+^/Na^+^ homeostasis and antioxidant defense in NaCl-stressed callus cells of two contrasting poplars. Plant Cell Tissue Organ. Cult..

